# Implementing computer‑aided detection for TB active case finding: A qualitative study of feasibility and acceptability in Tondo, Manila

**DOI:** 10.1371/journal.pgph.0007429

**Published:** 2026-10-08

**Authors:** Emelie Yonally Phillips, Ma. Danica Katrina P. Galvan, Juno Min, Cathy Hewison, Carmina Duyala, John Carlo Palmado, Sheryl N. Peral, Olivier Camelique, Mary Ruth C. Roxas, Marve D. Duka, Gina F. Pardilla, Maria Julieta C. Recidoro, Richard H. Castro, Farah Hossain, Helena Huerga, Valentina Carnimeo

**Affiliations:** 1 Epicentre, Interventional Epidemiology and Training Department, Paris, France; 2 Médecins Sans Frontières, Operational Center Paris, Tondo Project, Manila, Philippines; 3 Médecins Sans Frontières International, Amsterdam, Netherlands; 4 Médecins Sans Frontières, Operational Center Paris, Paris, France; 5 Research Office, Manila Health Department, Manila, Philippines; 6 TB Prevention and Control Section, Manila Health Department, Manila, Philippines; 7 Médecins Sans Frontières Operational Center Paris, Section Japan, Tokyo, Japan; University of Sydney, AUSTRALIA

## Abstract

Tuberculosis (TB) remains the leading cause of death from a single infectious agent, with over 1.23 million deaths globally in 2024. Despite available treatment and prevention strategies, timely and accurate detection remains challenging, particularly in high-burden settings such as the Philippines, which accounted for 6.8% of the global burden of TB in 2024. Computer-aided detection (CAD) software integrated with digital chest X-ray offers enhanced screening capacity, but its success depends on feasibility and acceptability within local health systems. This qualitative study explored perceptions and experiences of CAD integration during a Médecins Sans Frontières community-based TB active case finding campaign (ACF) in Tondo, Manila. In-depth interviews, focus group discussions, and direct observations were conducted with MSF staff, health center physicians, TB program personnel, ACF participants, and community leaders between January and April 2023. Thematic analysis identified four main themes: (1) Operational functionality and efficiency: Staff reported CAD increased screening capacity, supported clinical decision-making, and streamlined workloads; ACF participants reported a convenient process. (2) Human resources and role adaptation: Staff perceived role redistribution as initially challenging, highlighting the need for adequate capacity building and technical support; (3) Technical feasibility and system design: project managers and staff emphasized understanding CAD’s capabilities and limitations to plan infrastructure, set thresholds, and compensate for inherent limitations. (4) Community acceptability and ethical considerations: community participants expressed mixed views on the reliability of novel “high-tech” tools and stressed the importance of aligning with local healthcare needs. This study demonstrates that implementing CAD in TB ACF is both feasible and acceptable when supported by careful planning and adaptation to local contexts. Sustainable implementation hinges on technical support and the meaningful involvement of healthcare workers and communities, whose insights help ensure ethical and effective digital health innovation. The experience in Tondo offers lessons on deploying CAD in high-burden, resource-constrained settings.

## Introduction

Tuberculosis (TB) has been part of human history for thousands of years, with traces found in ancient remains from Egypt, Peru, and India [1]. Over time, its devastating impact earned nicknames like the “White Plague” and “Captain of All These Men’s Deaths” [1]. Since the discovery of *Mycobacterium tuberculosis* in 1882, TB has remained one of the deadliest infectious diseases [2]. Despite the availability of effective tools for prevention and treatment, TB continues to claim lives, an estimated 1.23 million in 2024 alone, making it once again the leading cause of death from a single infectious agent worldwide [3].

However, of the estimated 10.7 million people that fell ill with TB in 2024, approximately 2.4 million cases still went undiagnosed or unreported [3]. With almost 545 000 reported cases, the Philippines accounted for 6.8% of global TB cases and ranked among the countries with the largest gaps between newly notified cases and estimated incidence, highlighting significant challenges in case detection and reporting [3]. Factors such as TB’s varied clinical presentations, a high proportion of asymptomatic cases (50.4%) [4], inadequate diagnostic tools, and limited access to both diagnostic tools and qualified health care professionals in many areas, remain critical challenges for TB case detection [4,5]. Left untreated, TB has a case fatality rate close to 50% and contributes to ongoing transmission [6]. In the Philippines TB remains a leading cause of death, with an estimated 98 Filipinos dying each day from TB [7].

To address these challenges, there has been growing focus on active screening, also known as active case finding (ACF), to detect and treat TB early, improving both individual outcomes and reducing transmission [4]. ACF involves proactively screening individuals in contrast to waiting until individuals present to health facilities with symptoms for diagnosis, known as passive case finding. While traditional methods of ACF frequently rely on symptom screening, technological advancements are producing promising new tools for detection. Recent years have seen the validation of AI-based Computer-aided detection (CAD) software integrated with digital chest X-ray (CXR) to enhance TB screening [8,9]. CXR provides a highly sensitive screening method for detecting both symptomatic and asymptomatic cases of pulmonary TB [4,8–10], but is challenging to implement in many of the contexts on the WHO’s High Burden Countries for TB list [11] due to shortages of qualified radiologists to interpret the images. CAD software can be coupled with CXR to provide an automated interpretation of the digital image for TB detection, which independent reviews have validated as comparable to interpretation by experienced human readers [8–10].

However, in a context of widening health inequities, unstable global health financing, and rapidly expanding reliance on artificial intelligence, there is growing concern that digital innovations may exacerbate disparities if implemented without adequate attention to feasibility, acceptability, and ethical considerations within local health systems [12]. When introducing new healthcare technologies, particularly those involving artificial intelligence (AI), successful integration requires more than technical validation. It demands careful consideration of how these tools fit within existing health systems, logistical and infrastructural constraints, and the capacities of those responsible for their deployment and use [10,13,14]. In qualitative studies with clinicians, participants reported that AI can reduce workload and improve diagnostic reliability, but they still emphasized alignment with clinical needs, seamless workflow integration, training programs, and concerns about overreliance and skill erosion [15–17]. Patient-focused studies likewise show conditional acceptance, with participants reporting a preference for AI as a support tool under human oversight rather than as an autonomous decision-maker, and concerns about privacy, transparency, and the preservation of human attention and empathy [18–21]. Taken together, these findings support the need to understand healthcare workers, planners, and communities when designing AI systems so they remain relevant, usable, and trusted in local health-system context. Their insights help identify practical barriers to adoption while also highlighting opportunities to improve engagement and sustainability [10,13,14]. Without such input, even well-designed technologies risk being underutilized or misaligned with the realities of implementation [10,13,14]. While there is a growing body of research on CAD, most studies focus on performance. We found one study which qualitatively explored the perspectives of early implementers of CAD in various contexts [10]. However, at the time of writing, we are unaware of any research exploring patient perspectives of and experiences with CAD or healthcare worker perspectives and experiences implementing CAD in the context of the Philippines. This study assessed the acceptability and feasibility of implementing CAD within ACF activities led by Médecins Sans Frontières (MSF) in Tondo, Manila.

## Materials and methods

### Setting

The study was conducted in Tondo, Manila, a densely populated urban setting with substantial socioeconomic vulnerability and a high burden of TB. The National Capital Region, which includes Manila, consistently accounts for a large proportion of nationally notified TB cases. Tondo is home to more than 650,000 inhabitants, with population densities exceeding 69,000 people per km^2^ and high levels of poverty [22], conditions that contribute to elevated TB transmission risk and support the use of active case-finding approaches. In collaboration with the Manila Health Department (MHD), MSF launched community based ACF in Tondo in May 2022 targeting various community sites across District 1 of Tondo.

MSF collaborated closely with local health centers and Barangay councils (a barangay is the smallest local political unit in the Philippines) to facilitate outreach and engagement at various community sites. The MSF ACF team comprised nurses, radiologists, radiology technicians, and TB physicians who carried out symptom screening, CXR examinations using a digital X-ray machine housed in a mobile truck, clinical assessments, and sputum collection. Community members over 15 years old were invited to participate in ACF screening activities. Individuals who screened positive via symptoms or by CXR interpretation by a radiologist were asked to provide a sputum sample which was transported for diagnostic testing with GeneXpert in local laboratories. A TB physician further evaluated a subset of ACF participants. ACF participants with either bacteriologically confirmed or unconfirmed (clinically-radiologically diagnosed) TB were referred to their respective local health centers for treatment and follow-up.

In October 2022, MSF ACF staff and the Physicians in Charge from local health centers within the district received training and sensitization on the use and functionality of the CAD software, CAD4TB v.7 (Delft Imaging, Netherlands) for TB screening. The software was subsequently integrated into ACF activities in November 2022 to support screening, referral, and diagnostic triage. Rather than relying solely on a manufacturer-recommended threshold, a local calibration protocol was used to determine the CAD threshold for referral. An initial threshold was selected based on programme needs and referral patterns and was subsequently reviewed and adjusted during implementation using operational data. A detailed description of the threshold calibration methodology, programme outcomes, including screening volume, CAD-based referrals, and TB diagnoses, are reported elsewhere [23]. With the implementation of CAD, the patient circuit was updated. All ACF participants underwent symptom screening and CXR with CAD reading. Those who either screened positive for symptoms or had a CAD score above threshold were sent to the sputum collection station. While prior to CAD implementation, all CXRs were read by the radiologist, in the new patient circuit only CXRs with CAD scores above threshold were read by the radiologist. ACF participants who screened negative for symptoms and had a CAD score below threshold were directed to the exit registrar.

### Design, population and procedures

Between January 25^th^ and April 12^th^ 2023, we conducted a qualitative study focused on the perspectives of local community members, healthcare professionals, and managers.

Inclusion in the study was restricted to adults aged 18 years or older who were either community leaders, community participants in the ACF screening activities, or physicians affiliated with health centers in the Tondo district targeted by MSF ACF activities, as well as staff members of the MHD Tuberculosis Control Program or MSF project managers and staff. All participants were required to provide written informed consent for participation in the study and audio recording.

We conducted 31 in-depth interviews (IDIs), 3 focus group discussions (FGDs), and 4 direct observations. IDIs were conducted with the following groups: MSF ACF project managers and staff to explore their perspectives and experiences of planning and implementing CAD into the Tondo ACF project; Physicians-in-Charge of the local health centres (HC) and MHD TB Control Program staff to assess the feasibility and acceptability of CAD integration, particularly in terms of alignment with local and national health systems processes; and ACF participants to understand their personal experiences of undergoing TB screening in the context of Tondo using CAD and CXR. FGDs were conducted with community leaders in the barangays where ACF was implemented to understand broader social dynamics, community perspectives and experiences related to TB, the MSF ACF activities, and the use of CAD and CXR for TB screening. Direct observations lasting one hour were conducted at the ACF sites to understand the patient circuit, observe the environment and set-up, and document flow and turnaround times.

### Sampling

Non-probability sampling methods were used to identify participants. Community leaders were identified by the Barangay Chairperson and recruited to participate in the FGDs. Purposive quota sampling was used to identify ACF participants in person at the end of screening by the exit registrar nurse using quota of gender and CAD score above and below threshold, with efforts made to include ACF participants of various ages and those recruited to the ACF site through community mobilization and household contact tracing. The Physicians-in-Charge of the four HCs in which MSF ACF activities had been conducted at the time of the study were recruited into the study in person. Purposive sampling was used to identify members of the MSF Tondo project managers and staff and the MHD TB Control Program that would be able to provide the most information about the acceptability and feasibility of implementing CAD into this project and context.

### Interpretivist orientation and theoretical framework

The study was informed by an interpretivist orientation, recognising that participants’ experiences and perceptions of CAD implementation are shaped by their professional roles and contextual realities. This approach underpinned the use of qualitative data collection methods and thematic analysis. To provide a structured framework for understanding feasibility and acceptance of the implementation of CAD into MSF’s ACF activities in Tondo, we have employed the Unified Theory of Acceptance and Use of Technology (UTAUT) [6]. UTAUT posits that the acceptance and use of technology is informed by performance expectancy, effort expectancy, social influence and facilitating conditions. Gender, age, experience, and voluntariness of use are also incorporated into the UTAUT as potential influencers (Fig 1). Semi-structured FGD and IDI guides tailored to each study participant group were developed to explore each aspect of the UTAUT framework (S1 Tools).

**Fig 1:**
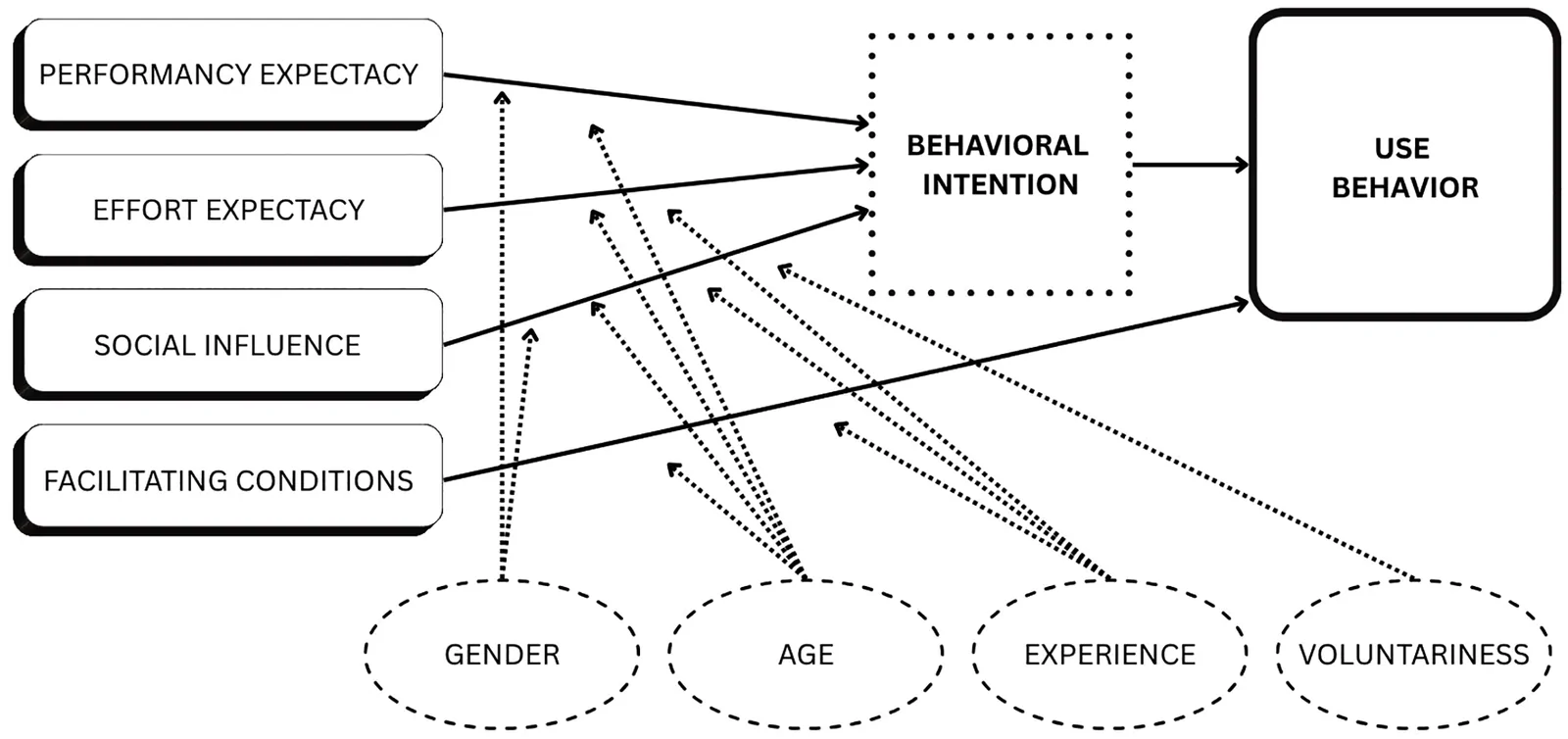
The unified theory of acceptance and use of technology [6].

### Ethics and positionality statement

The study protocol received approval from the University of the Philippines Manila Research Ethics Board (UPMREB) (Ref.: UPMREB 2022-0310-01) and the MSF Ethic Review Board (Ref. ID: 2239). The study was designed and conducted in accordance with the principles of biomedical research involving human subjects of the Helsinki Declaration (2013) developed by the World Medical Association Declaration. An informed consent process was carried out with each study participant and written informed consent obtained prior to inclusion in the study.

The qualitative study was led by E. Yonally Phillips, MPH, a senior mixed-methods researcher with Epicentre, with oversight by V. Carnimeo, MsC, MPH, a senior epidemiologist at Epicentre, and the Principal Investigator of the study. Local researchers with contemporary ties to the Philippines, J.C. Palmado, C. Duyala, and S. Peral, were recruited by MSF to collaborate in tool refinement, data collection, transcription, and analysis. All of the research team received theoretical and practical training in the qualitative methods, research ethics, and the study objectives, methods, and tools. The research team also worked together to revise and translate the tools, ensuring they were relevant and adapted to the local context.

In the context of Tondo, decisions about interviewer assignment were made to minimize power imbalances and ensure participant comfort. EYP, an American with contemporary ties to Western Europe, conducted interviews with MSF healthcare staff, Physicians-in-Charge of HCs, and National TB Program staff. JCP and CD led activities with community leaders and ACF participants, with gender matching applied where appropriate to foster trust and comfort.

We acknowledge that our identities and affiliations may have influenced interactions and interpretation. To mitigate bias, we engaged in reflexive practices, including team discussions on assumptions, end of data collection day debriefs, and triangulation of data sources, to ensure findings reflect participants’ perspectives.

### Data collection

All tools were pilot tested prior to data collection. The qualitative research team conducted pilots in a barangay that was not included in the study in which ACF activities had been conducted prior to the implementation of CAD. A pilot FGD was held with community leaders and two pilot ACF participant IDIs conducted with community members. The IDIs for MSF staff, Physicians-in-Charge, and MHD staff were piloted with MSF project staff that were not recruited into the study.

FGDs and IDIs were conducted either in Filipino or English, depending on the preferences of the participants and audio recorded. FGDs had a duration of up to 90 minutes, while IDIs lasted no longer than one hour.

FGDs with community leaders were conducted by CD and JCP, with one acting as facilitator and the other as note-taker, in the local barangay community centre. IDIs were conducted with one participant and one researcher. ACF participants’ IDIs were conducted directly after screening by CD and JCP with researcher and participant matched by gender. These IDIs were conducted at the ACF site in private tents. Two IDIs with Physicians-in-Charge were conducted in their offices at their respective health centers. The other two were conducted later via Zoom meeting after the ACF activities had been conducted in their catchment areas. IDIs with MSF staff took place in the MSF project offices and IDIs with MHD staff took place in the Manila TB Control office.

Directly following each FGDs and IDIs, field notes were written by the researcher who conducted the activity to capture emerging themes, record observations of the setting and interaction, and monitor data saturation.

Four direct observations, each lasting one hour, of the ACF site were conducted in two different ACF locations and at various times of the day. Data collection took place between the end of January and April 2023.

### Data analysis

During daily debriefings with the research team, preliminary findings were discussed. EYP, JCP, and CD each typed pre-liminary findings and themes for each UTAUT component at the close of the day. After data collection, all audio-recordings were transcribed verbatim by SP. Filipino transcripts were then translated into English and a list of colloquialisms and slang definitions created to accompany the translated transcripts. EYP then conducted thematic analysis using NVIVO software on all transcripts, field notes, and observations to identify patterns, concepts, and themes. A combination of inductive and deductive approaches was used in the analysis. Initially all qualitative data were analysed with inductive open coding to explore and consider all possible meanings, and an initial coding scheme was developed. Following this open, descriptive coding, codes were organized into sub and parent-codes. Parent codes were further grouped into themes connected to the relevant components of the UTAUT framework and checked against the pre-liminary findings and themes noted by JCP and CD. Final results were shared with JCP and CD for review and validation.

## Results

From the perspective of healthcare planning and provision, IDIs were conducted with eight MSF project managers and staff including the Medical Team Leader, ACF Supervisor, Radiology Technician, Radiologists, TB Doctor, Project Coordinator, and Logistics Coordinator. IDIs were also held with four Physicians-in-Charge from the HCs where ACF activities had occurred and three members of the MHD TB control programme.

A total of 35 community members from 5 barangays in Tondo participated in the study, including participants in three FGDs with 19 community leaders (n = 11 men, n = 8 women) and 16 IDIs with ACF participants (n = 8 women, n = 7 men and n = 1 identified as gender nonconforming). Participant socio-demographics are illustrated in Table 1. Of the 16 patient participants, half had CAD scores above the threshold on screening (n = 8).

**Table 1:** Community participant demographics.

	ACF Participant IDIs	Community Leader FGDs	Total
Total	**16**	**19**	**35**
**YEARS OF EDUCATION**			
Average years (range)	8.0 (0-15)	9.3 (6-12)	8.7 (0-15)
**PERSONS IN PARTICIPANT HOUSEHOLD**			
Average number (range)	6.2 (3-13)	7.7 (1-17)	7 (1-17)
**PARTICIPANT LOCATION BY BARANGAY NUMBER**			
105	6	–	6
125	6	5	11
135	2	–	2
138	–	14	14
146	2	–	2

Thematic analysis of the interviews and group discussions identified four overarching themes related to feasibility and acceptability of CAD implementation: operational functionality and efficiency, human resources and role adaptation, technical feasibility and system design, and community acceptance and ethical considerations. Each of these overarching themes encompasses multiple sub-themes which reflect various components and combinations of components of the UTAUT. Table 2 provides a conceptual mapping of the identified themes and sub-themes to the UTAUT domains that informed their interpretation.

**Table 2:** Feasibility and Acceptability of the implementation of CAD, emergent themes and associated UTAUT framework components.

Theme	Sub-theme	UTAUT Component(s)	Participants
Theme 1. Operational Functionality and Efficiency	1.1 CAD should be used and understood as a tool for screening only	Performance Expectancy	MSF Staff
	1.2 CAD increases screening capacity	Performance Expectancy	MSF Staff
	1.3 CAD provides support for clinical decision-making	Performance Expectancy	MSF Staff
	1.4 CAD streamlines workload	Effort Expectancy	MSF Staff
	1.5 CAD is an easy tool to use in the screening process	Effort Expectancy	MSF Staff
	1.6 The screening process with CAD is perceived as convenient by ACF participants	Effort Expectancy	ACF participants
Theme 2. Human Resources and Role Adaptation	2.1 Implementation of CAD results in a redistribution of roles and responsibilities	Performance Expectancy; Effort Expectancy; Social Influence	MSF Staff
	2.2 Implementation of CAD necessitates capacity building and technical support	Effort Expectancy; Facilitating Conditions	MSF Staff
Theme 3. Technical Feasibility and System Design	3.1 Understanding and planning the necessary technical infrastructure to implement CAD is essential	Performance Expectancy; Facilitating Conditions	MSF Staff
	3.2 Screening process must compensate for inherent software limitations	Performance Expectancy; Facilitating Conditions	MSF Staff
	3.3 Outcome expectations must be balanced with establishing a feasible threshold	Performance Expectancy; Facilitating Conditions	MSF Staff
Theme 4. Community Acceptability and Ethical Considerations	4.1 Perceived accuracy and reliability of ‘high-tech’ is nuanced	Performance Expectancy	ACF participants; Community leaders
	4.2 Alignment with community healthcare needs, priorities and expectations	Performance Expectancy; Effort Expectancy; Social Influence	ACF participants; Community leaders
	4.3 Social obligations and decision-making around TB screening	Social Influence	ACF participants; Community leaders

### Theme 1: Operational Functionality and Efficiency

#### 1.1. CAD should be used and understood as a tool for screening only.

MSF staff emphasized that CAD’s role, like the role of CXR in general, in TB activities must be understood as a tool for screening, not diagnosis. While screening through CXR has been validated and is perceived as highly useful for identifying pulmonary TB, clinical and laboratory diagnostics were both noted as essential follow-up steps. As one participant explained,

*“You still have to confirm it with Xpert and you still have to correlate that. And the thing is, with CAD, and in any X-ray, any imaging actually... there’s always that little phrase at the end of the report that says, please correlate clinically.” – MSF staff*

While MSF staff thought that CAD could be remarkably useful in under-resourced settings and challenging contexts, they cautioned that it should not be relied on alone for TB diagnosis.

#### 1.2. CAD increases screening capacity.

All MSF staff reported that the implementation of CAD increased the screening capacity of the ACF site. This was attributed to two observed changes after implementing CAD. First, the rapidity of CAD results compared to a human reader significantly reduced the time to receive chest X-ray interpretations. Second, modifications to the patient circuit enabled by the use of CAD allowed time to be spent more efficiently, since ACF participants with negative symptom screenings and CAD scores below the threshold could proceed directly to the exit registrar. This reduced bottlenecks at the radiologist station and allowed staff to focus on ACF participants more likely to need care. As one participant described,

*“Basically, with the screening process more efficient, we can accommodate more patients in a shorter amount of time.” – MSF staff*

#### 1.3. CAD provides support for clinical decision-making.

MSF staff perceived CAD as providing support for clinical decision making for both the radiologists and the TB doctor on site. These participants described the software as providing a useful ‘second opinion’. When the CAD score, heatmap, and healthcare worker’s assessment aligned, the CAD outputs provided confirmation that increased the healthcare worker’s confidence in their decision, particularly for more challenging cases. When the CAD outputs and healthcare worker assessment differed, this was described as prompting a more thorough examination on the part of the healthcare worker. The heatmap output of CAD was additionally noted as helpful for identifying abnormalities on the patient’s X-rays, for both those with specialized and more basic X-ray interpretation skills. As one participant explained,

*“CAD is very didactic, very easy to follow. So even if you don’t have much experience with TB, you will see so much, so when you are taking clinical decision about whether to treat or not the patients, then it’s a very good assistance.” – MSF staff*

#### 1.4. CAD streamlines workload.

MSF staff reported that the CAD software significantly reduced the administrative workload on the team, as data that had previously been recorded manually could now be extracted automatically from CAD. Additionally, CAD was noted for significantly reducing the workload for the radiologists by filtering out ACF participants with CAD scores below threshold and a very low likelihood of active pulmonary TB. With CAD introduced to the circuit, radiologists only needed to review CXRs and write reports for ACF participants who had a CAD score above threshold.

*Without CAD we were doing a lot of data entry for the chest X-ray. It was a requirement. It was then done manually, so it’s another administrative step in the circuit… It takes time. With CAD, everything is stitched up so we can get the logbooks from the CAD. – MSF staff*

#### 1.5. CAD is an easy tool to use in the screening process.

MSF staff also discussed the relatively easy screening process resulting from the implementation of CAD. Participants viewed the software itself and the interface as straightforward and easy to learn and use. The CAD outputs were described as simple, a heatmap on the CXR image highlighting TB findings and a numeric score from 0 to 100 indicating the overall likelihood of TB based on the imaging findings, with 0 being extremely unlikely and 100 being extremely likely. As one MSF staff described,

*There were literally just like two buttons I had to press with the interface. So, like, the learning curve was not very, very steep, pretty straightforward… In terms of using CAD… I definitely think having the whole chest X-ray reading course is very helpful because CAD will only give you colors and the number... that might need some training and experience and learning. – MSF staff*

However, Participants noted that the visual heat map and numerical CAD score did not always appear intuitively aligned, with some CXRs displaying markedly different heat map patterns despite receiving similar CAD scores. While this did not represent a technical error in the software, it created uncertainty among some users regarding how the outputs should be interpreted. Examples of CAD outputs illustrating this phenomenon are provided in Figs 2 and 3. In these instances, it was critical to reinforce that the CAD score was the most important output to guide referral decisions. Participants noted that the process of learning to accurately interpret the CAD outputs and integrate them into their own practice and decision-making was an iterative process of training, learning in the field, and discussing with the team.

**Fig 2:**
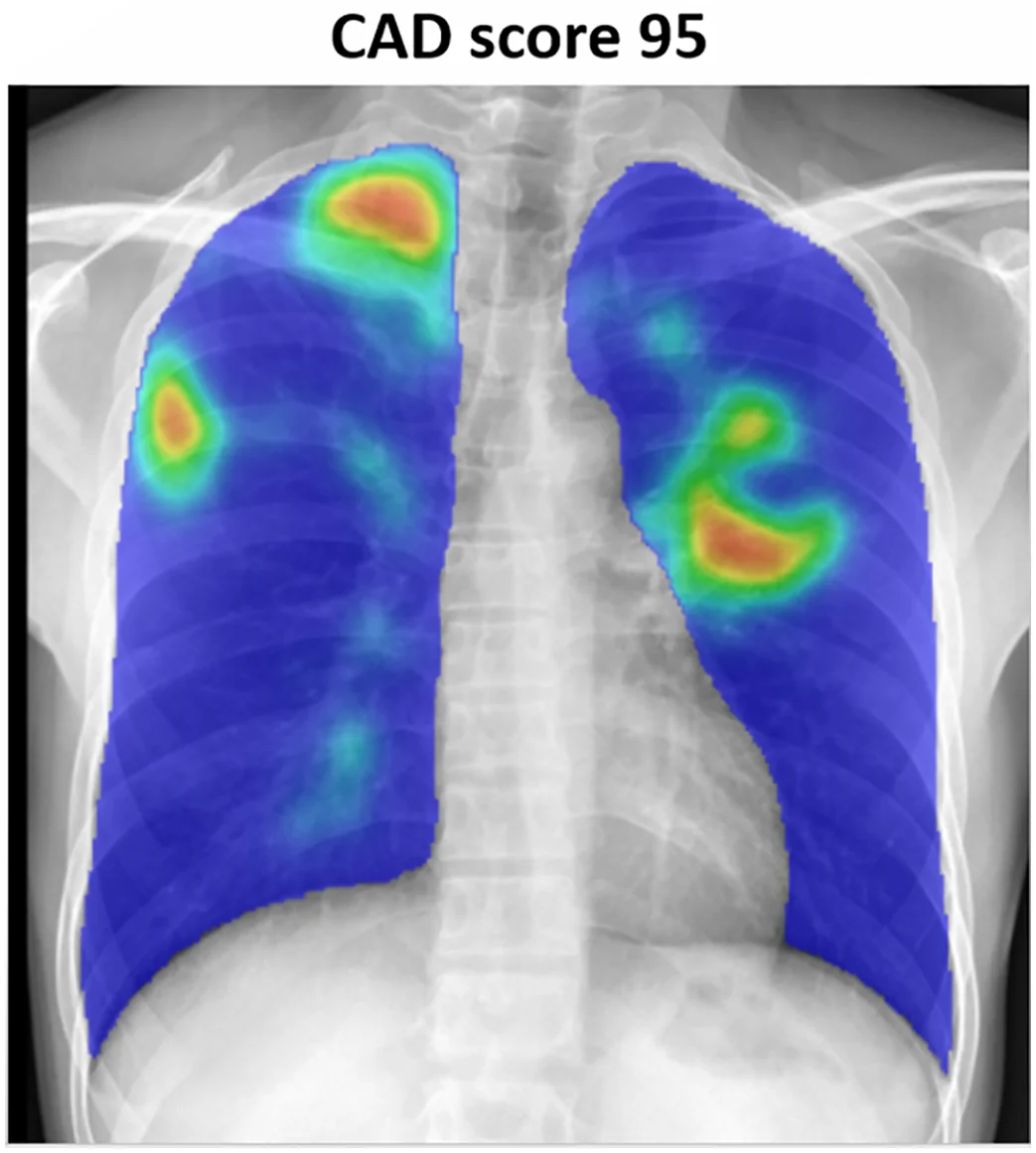
CAD heatmap output of a CXR with CAD score of 95.

**Fig 3:**
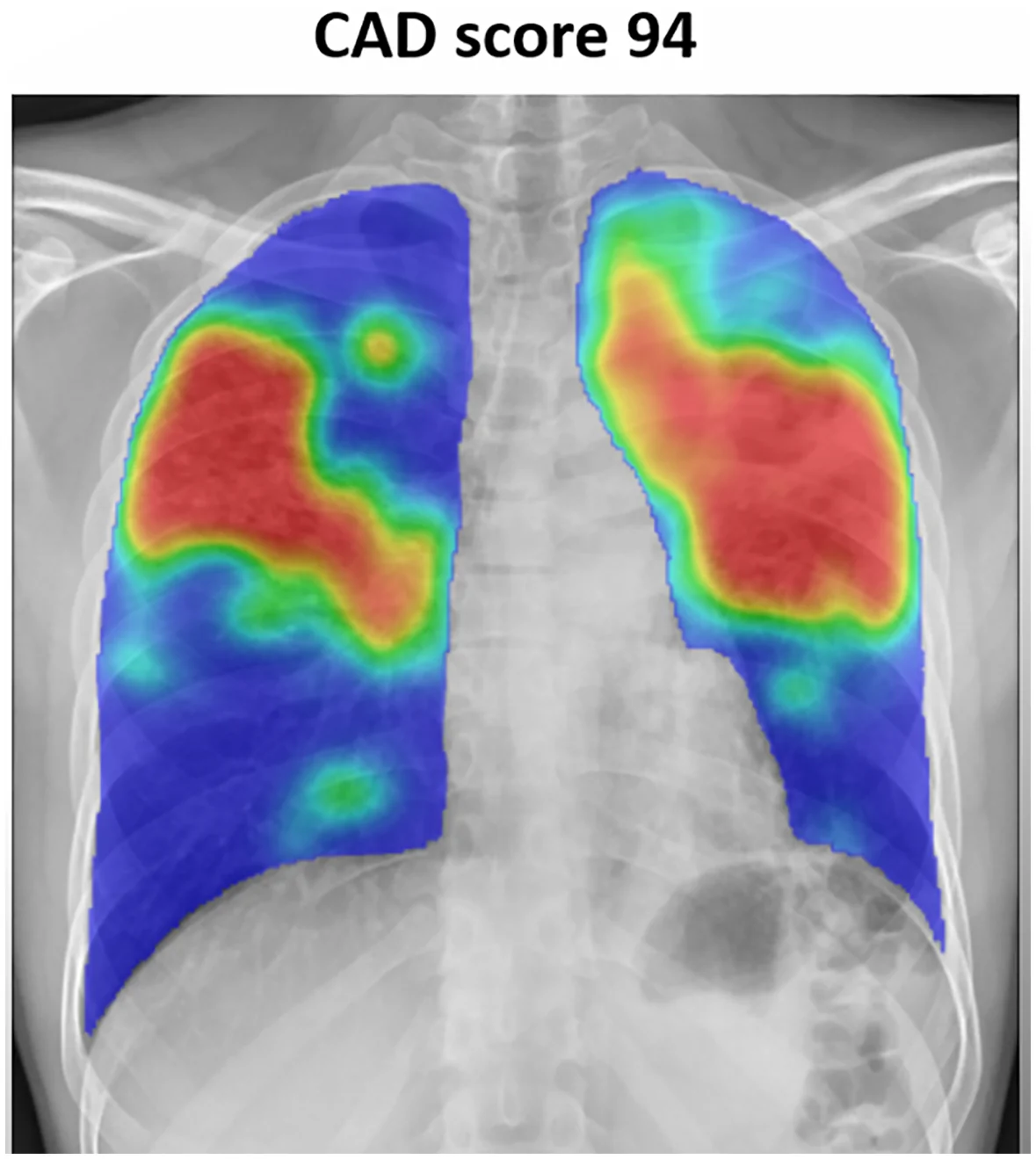
CAD heatmap output of a CXR with CAD score of 94.

#### 1.6. The screening process with CAD is perceived as convenient by ACF participants.

Although ACF participants had limited specific knowledge about CAD, many appreciated the convenience of the ‘high-tech’ X-ray readings, which eliminated long queues, referrals, dependence on doctor availability, and the need for return visits, resulting in an easier, more rapid and convenient patient experience. As one participant described,

*It’s high tech now, wherever you go. That’s actually better because it’s easier... it’s like a computer so it’s easy. It’s automatic, you can get the results right away... unlike before, it took long. - ACF participant, man*

Some ACF participants even described this easier and more rapid process as reducing the time individuals deal with fear and uncertainty and allowing for quicker treatment initiation. As one patient explained,

*It was a long process [before MSF ACF project], so how can you take medication immediately, if you have TB? Then, just here where it’s faster, so you can immediately take medicine. - ACF participant, woman*

However, ACF participant and community leaders did note that community members with severe illness, disability or mobility issues would still face challenges accessing the screening site or standing for a chest X-ray.

### Theme 2. Human Resources and Role Adaptation

#### 2.1. CAD implementation results in a redistribution of roles and responsibilities.

The integration of CAD led to a redistribution of roles and responsibilities. In terms of effort and performance expectancy, these changes were perceived as positive, resulting in reduced administrative workload, decreased demands on radiologists, and enabling staff to focus their efforts on ACF participants with higher likelihoods of requiring follow-up care. While participants did not specifically describe increased time spent in direct patient interactions, they reported that the reduction in administrative tasks allowed them to concentrate on activities more closely aligned with their clinical and programmatic responsibilities.

*Because if you remove administrative tasks then that’s what I mentioned that it will focus more on what you want, you need, really need to do. – MSF staff*

While these changes were mostly regarded by MSF staff as aligning with their perceived roles and expertise, a learning curve for interpreting the CAD outputs was noted for staff whose roles did not previously include examining X-rays.

*While I’m not a radiologist, I don’t necessarily have that training with an eye to say, oh, this is really, really bad. At least I can see, what’s involved, in a way. And the CAD score helps. – MSF staff*

However, changes to the decision-making structure that shifted the responsibility of referral for testing from the human reader to the CAD caused initial friction, particularly for those whose responsibility this decision had previously been. While it was acknowledged that overall, CAD and human readers had similar rates of referral for testing, case by case discrepancies created concerns. These staff members described still feeling social responsibility for the outcome, which resulted in them wanting the ability to override CAD results that they perceived as incorrect. They described a desire to ensure that potential TB cases were not missed by CAD, or conversely, that healthy individuals did not have to worry and undergo sputum collection unnecessarily and use limited resources due to a false positive CAD score.

*I think there’s only one thing that I would like to, you know, not change, but the power… to have the patient tested. When we see a…. the below threshold ones. We would like to have it tested so it can be confirmed. Yeah, but it uses resources, so maybe not. – MSF staff*

Radiologists also noted that in cases where ACF participants X-rays were above CAD threshold, but the radiologist thought the X-ray looked normal, this put them in the challenging position of having to write a report for findings they did not agree with which was described as compromising their responsibilities.

*When it’s above threshold, but I think it’s normal, so, that’s also a challenge because how can I put the… what findings will I put, specific findings that I put regarding the image if I think that it’s normal? So, I kinda sometimes I compromise, like […] if the CAD or software is saying that this kind of lesion and then I just check it [on the form] (laughs) even though I don’t agree. – MSF staff*

#### 2.2. Implementation of CAD necessitates capacity building and technical support.

MSF staff described that while the CAD software itself was straightforward to install, the design and initial set-up necessitated a lot of capacity building and technical support. Prior to implementing CAD, the data management and storage system needed to be designed in line with the software’s technical requirements and national and international data management and confidentiality guidelines. This required a substantial amount of technical expertise and consultation. As one of the MSF mission staff described,

*Internally we, we discuss a lot about… where will the images be stored? Will it be in the cloud? I mean, is the cloud secure? Can we ask someone, so we always have experts weighing in on is this allowed? Will, will… no one can hack into it? – MSF staff*

Additionally, the implementation of CAD requires the selection of a threshold score, above which ACF participants are referred for confirmatory testing. The threshold selection should be customized to the local context, taking into consideration factors such as the characteristics of the population, screening objectives, and laboratory testing capacity. Establishing an acceptable and feasible threshold, adjusting the threshold as needed, and redesigning the patient circuit to include CAD and align with Ministry of Health guidelines for TB screening required a substantial amount of technical knowledge and support.

*The initial setting up was difficult in the sense that, like, setting a threshold even in the first place was difficult. So, we definitely saw a lot of technical assistance, trying to troubleshoot CAD, trying to figure out how to best [implement CAD]. – MSF staff*

MSF staff emphasized the need to consider the technical expertise of the team and the need for additional specialised technical support. The job descriptions should be carefully considered for a project involving CAD implementation to ensure that the set-up of the system and adjustments and troubleshooting can be well supported.

### Theme 3. Technical Feasibility and System Design

#### 3.1. Understanding and planning the necessary technical infrastructure to implement CAD is essential.

MSF staff noted that implementation of CAD requires understanding its operational prerequisites and planning accordingly. CAD implementation requires consistently high-quality digital X-rays, as image quality can affect the CAD results. Also, appropriate threshold calibration is specific to the local conditions. For example, participants said that ideally the CAD software would perform analysis on images from the same type of X-ray machine that was used to select the initial threshold. As one MSF staff member explained,

*If you define a threshold for the CAD box with X-ray, if for any reason, you use another X-ray to provide the images, if it’s not exactly the same model and the same quality of images, you cannot use your threshold. – MSF staff*

Additionally, it is also necessary to have staff on the project with the knowledge and skills to obtain high-quality X-rays. As one participant noted,

*CAD scores are very dependent on the quality of the chest X-rays taken. So, if… if the patient of the X-ray was taken in poor inspiration… CAD tends to have a false positive result. – MSF staff*

#### 3.2. Screening process must compensate for inherent limitations of CAD.

MSF staff emphasized that realizing the full potential of CAD requires a solid understanding of its inherent limitations to ensure effective integration and appropriate program design adaptations. CAD is recommended by WHO only for screening and triage for pulmonary TB in individuals aged 15 and older [24]. CAD is not validated for use in children and cannot reliably identify typical radiological signs of TB in children, so a human reader is needed to successfully screen this population with chest X-ray. As one participant explained,

*The typical finding you would see on a kids’ chest X-ray, that’s highly likely for TB, it’s your, its bigger lymph nodes, it’s not something CAD seems to be picking up. So, on an X-ray, this is a kid with very high, very high likelihood of TB, with CAD, would miss it entirely because it’s not something that’s going to pick up. – MSF staff*

Users further described patterns in CAD scores suggesting that the software has a tendency to give inflated scores to older ACF participants with a history of TB or other lung conditions, has challenges distinguishing prominent nipple shadows if unmarked, and has challenges distinguishing between lung opacities and soft tissues of the neck. As one MSF staff noted,

*Because I found that the CAD score varies a bit, with age. In the sense that people who are older would have CAD scores in the 40s and 50s and those might, those people might not actually have TB. It’s just that they’ve had like a lot of other things going on, like other previous lung diseases, that might just raise the CAD score of that. – MSF staff*

Staff participants reported that as they gained a better understanding of the capabilities and limitations of CAD, they were able to adapt the screening program to integrate CAD more effectively, leveraging its strengths and compensating for its weaknesses with other components. This was noted as an important consideration for other programs wanting to implement CAD.

#### 3.3. Outcome expectations must be balanced with establishing a feasible threshold.

MSF staff highlighted the tension between the expectations of a highly sensitive CAD reading and the need to set a CAD threshold that resulted in a feasible number of referrals for diagnostic testing. MSF project staff understandably wanted a screening process that would catch every case of TB. However, while a lower threshold would be more sensitive, it would also result in a higher number of ACF participants referred to GeneXpert testing. The number of feasible referrals was limited by the local GeneXpert laboratory capacity and the need for a rapid turn-around time of results for the highly mobile population. If the laboratory capacity was overwhelmed, the significant increase in turnaround times for results was described as having a negative impact on patient follow-up care and treatment initiation. Selecting a threshold that balanced staff expectations for sensitivity and resulted in a feasible number of ACF participants referred to GeneXpert testing was described as the ‘sweet spot’ by multiple MSF staff.

*There’s a sweet spot for the threshold, so they, they just need to find it out. So that, you know the level of sensitivity and specificity… If they can find that sweet spot, it will be very, the CAD box will be very, very good. – MSF staff*

Further, this ‘sweet spot’ was described as mutable and dependent on the number of working GeneXpert modules and the number of ACF participants accessing the ACF site.

*And I don’t know when we’re gonna need to adjust the threshold again. But I’m not sure if the current threshold is working with the numbers that we’re finally pushing with the ACF group. We’re finally doing triple digits and I don’t know if we’re overloading Xpert versus before when we had less patients. So that could have impacted flow. – MSF staff*

### Theme 4. Community Acceptability and Ethical Considerations

#### 4.1. Perceived accuracy and reliability of ‘high-tech’ is nuanced.

While patient participants reported little knowledge or understanding of CAD, most described that the use of ‘high-tech’ screening software improves the accuracy of the outcomes. As one patient expressed,

*This is better because it’s already computerized, it doesn’t make mistakes. It’s not like people who blink their eyes. - ACF participant, man*

However, some of the community and patient participants raised concerns about the reliability of a newer, untested technology. Elderly community members were also perceived by some as more likely to trust the diagnosis of a doctor over AI software. Additionally, ACF participants who received CAD results that were inconsistent with the symptom screen or GeneXpert test results reported confusion and some hesitancy over the reliability of the results. As one patient expressed,

*The X-ray was negative, the sputum was positive. The two are not consistent. - ACF participant, woman*

#### 4.2. Alignment with community healthcare needs, priorities and expectations.

Community participants also raised the issue of how well the TB screening aligned with the broader needs, priorities, and expectations of the community. In a context with limited resources, community members have many competing priorities to secure the health and well-being of themselves and their families. As the screening was only focused on TB, some participants highlighted the relative advantage of going to the health center to have a more comprehensive health assessment. As one patient explained,

*I’d rather have a doctor so I can figure out how I feel. What my illness is. - ACF participant, man*

Some patient responses indicated a mistaken understanding that CAD performed a comprehensive health check and that scoring below the set CAD threshold meant that the patient was disease free. As one participant is quoted,

*It’s computerized already, that’s why it can detect all your diseases. - ACF participant, man*

Additionally, some ACF participants explained that their motivation for coming to the screening was to obtain a TB screening report that would clear them for work. However, it was unclear if the CAD report created by MSF and given to ACF participants would meet workplace requirements or if it would be better to go to a hospital or private clinic for the required screening. As one patient stated,

*This X-ray... can be used for work. That’s why I asked if I could use it; they said it was possible. - ACF participant, man*

#### 4.3. Social obligations and decision-making around TB screening.

Community members discussed social influences not in terms of acceptance of CAD or chest X-rays, but in terms of decisions to undergo TB screening. Community members described complex social norms relating to stigma and embarrassment around TB. Some indicated that the social avoidance of individuals with TB was a demotivator for undergoing screening, while others described social responsibility as a driver of TB screening and treatment. Once someone in the community is diagnosed, community participants reported that TB patients are encouraged by family and neighbors to go for check-ups and adhere to treatment to prevent possible transmission to others. As one patient expressed,

*This is why I went for check-up, because I kept on coughing, for 2 weeks now. So my housemates won’t get infected. That’s why I went here. - ACF participant, man*

ACF participants explained that decisions about TB screening are also shaped by practical dependencies, with those who bear the cost and responsibility for taking the patient to the health center often making the final decision. One patient noted that the ACF activity was free and offered direct invitations, which empowered her to decide independently to participate.

*If I’m the one feeling ill, and needed to go for check-up, then you can suggest to your son or children that you’re ill and needed to see a doctor […] but when it comes to that, like free stuff, I make that decision. […] in events where there are invitations, I made the decision because I want to be treated. - ACF participant, woman*

## Discussion

This qualitative study explored MSF staff, health workers, and community members’ perceptions of feasibility and acceptability of CAD integration into a TB ACF project. The findings suggest that both feasibility and acceptability were negotiated processes. Project staff and ACF participants perceived gains in screening performance and efficiency, including increased screening capacity, optimization of the screening process, and support for clinical decision making. However, achieving these gains required careful consideration of the local context and project resources and objectives. Participants highlighted that implementation of CAD necessitated infrastructural and technical planning to support the CAD software and integrate into the local healthcare system, and investments in capacity building and training to foster competencies and confidence. This interpretation is consistent with qualitative research in other clinical settings, where clinicians described acceptance of AI as dependent on trust, clinical integration, training, and the technology’s fit with professional practice rather than on technical capability alone [15,16]. Additionally, this study highlights how community participation in TB screening using CXR with CAD was shaped by perceived healthcare needs, trust in advanced technology, and the simplicity of the process. However, misconceptions about CAD’s capabilities underscore the importance of clear communication and community engagement for maintaining trust.

Implementation of CAD in the MSF ACF project was described as improving screening performance and efficiency through yield maximization, process optimization, and capacity creation. Participants perceived that CAD substantially increased the screening capacity of the project by reducing turn-around time for results and by using CAD scores to triage referrals to GeneXpert testing and radiologist review. According to participants, this effectively reduced the proportion of CXR images requiring radiologist review and increased overall screening capacity without overburdening the radiologist or contributing to concerns about reader fatigue. The associated reduction in total time individuals spent in the screening circuit, particularly for those who screened negative and could proceed directly to the exit registrar after symptom screening and CAD reading of their CXR image, may have also improved community participation, as screened community members described the process as ‘fast’ and ‘easy’.

Further, while in this case CAD did not act as a complete replacement for a human reader, it did create capacity in the form of ‘allocative efficiency of human reader time’ [14]. By reducing administrative workload through the electronic recording of patient data and triaging individuals unlikely to have TB out of the circuit, CAD allowed project staff to reallocate their time and focus to cases most likely to need attention. In the instances that ACF participants were referred to the radiologist or TB doctor on site, users noted that the CAD outputs provided a useful ‘second opinion’, either confirming their assessment or prompting a more thorough examination if interpretations differed. Staff perceived this support for clinical decision making as improving performance. Staff further noted that in areas where shortages in human readers present major barriers to TB detection, using CAD in place of radiologists has the potential for even greater impact [14]. However, while CAD can provide automated CXR interpretation for TB screening, there is a significant risk of overreliance and use beyond its recommended purpose, for instance, to confirm TB diagnosis, to diagnose non-TB conditions and for persons under 15 years of age [25].

The initial implementation of CAD into the ACF circuit required specialized technical support for both installation of the software and creation of a cloud storage system that adhered to data management and confidentiality ethics [9]. The mass amounts of data generated by screening projects may necessitate cloud storage solutions, which require infrastructure, national policies, and political sensitivity to these issues. This can pose difficulties for national programs and contribute to ongoing political dependencies on technologies and the donors associated with them [26].

Additionally, the performance of the CAD software was reportedly dependent on CXR image quality. For projects implementing CAD in areas with gaps in human resources, it should be noted that ideally a trained radiographer would obtain high-quality CXR images for a TB screening program. Further, as CAD is currently only recommended for TB screening for individuals 15 years and above, projects implementing CAD must carefully consider alternative options for children and young adolescents [27].

Due to substantial variance in diagnostic accuracy across contexts, standardizing thresholds is not feasible [10]. Thresholds must be calibrated to local conditions, including population characteristics [10,24]. In this project, a pragmatic approach was used by selecting an initial threshold and adjusting it based on programmatic data, diagnostic capacity, and patient flow and is described in detail elsewhere [23]. As both diagnostic capacities and community participation shift over time, CAD demonstrates the advantage of providing consistent results that can be rapidly adjusted to a changing context [14]. This reactive adjustment capability also improved staff confidence, which initially suffered from cases in which the CAD score and on-site radiologists’ readings diverged. Ongoing reviews of project data, particularly positivity rates at different CAD scores, and reactive threshold adjustments helped build trust in CADs performance and staff’s ability to maintain quality oversight and adjust as needed [26]. These findings also reflect a broader pattern in qualitative patient and practitioner research showing that acceptance is generally stronger when AI is positioned as a support tool under human oversight rather than as an independent decision-maker [16,28,29].

Once installed and established in the ACF circuit, the CAD software was reportedly simple to use and had a user-friendly interface. However, interpreting the CAD outputs was not intuitive, and initial misconceptions about the CAD score and heatmap resulted in confusion (for example, a low CAD score accompanied by substantial heatmap markings on the image) [26]. While the heatmap provides a visual representation of the CXR abnormalities, the CAD score indicates the likelihood of TB and should be used for triage decisions [26]. Training sessions on the functionality, capacities and limitations of CAD, along with in-depth, hands-on training were noted for improving staff understanding of what the CAD outputs represent and how to interpret them accurately. Other qualitative studies of clinician-facing AI similarly identify training and co-design as central to safe adoption [15–17,30]. These training sessions further improved staff confidence in CAD’s performance and in their own ability to utilize the software to maximize benefits and compensate for limitations through checks and balances in the project design. Having this deeper understanding of CAD was described as useful for designing an optimized patient circuit, determining project monitoring data metrics, and adjusting the threshold to meet screening targets. Projects implementing CAD in areas with less availability of qualified HR may consider how to invest more in initial training and capacity building to ensure success.

Community acceptance and participation in TB screening with CXR and CAD were shaped by perceived need, the appeal of high-tech accuracy, and the simplicity and speed of the screening process. In the context of an underserved neighborhood with limited access to radiological services, the availability of CXR met a significant demand for accessible healthcare. Additionally, the high burden of TB and employment requirements for negative TB screenings further motivated participation. ACF participants generally trusted the CAD technology, often associating it with advanced capabilities, even if they were not fully aware of its specific functions. This aligns with hypotheses that TB screening with CAD may contribute to higher community participation if perceived to be ‘cutting edge technology that they could not otherwise access’ [14]. However, discussions with ACF participants revealed several misconceptions, including beliefs that CAD could detect all diseases, leading to unrealistic expectations. Confusion also arose when CAD results conflicted with symptom screening or GeneXpert outcomes, highlighting the need for clear communication and expectation setting. Some participants expressed a preference for broader health assessments rather than TB-only screening, suggesting that while CAD contributes to acceptance, its implementation must be accompanied by robust community engagement to prevent misunderstandings that could erode trust.

### Limitations

While this case study provides valuable insights into the feasibility and acceptability of implementing CAD within an active TB case‑finding project in Tondo, Manila, several limitations should be considered.

First, data were collected at a single time point, approximately three months after CAD implementation. This timing was selected to balance the need to capture participants’ early implementation experiences while allowing sufficient time for adaptation, learning, and integration into routine practice. Nevertheless, the cross‑sectional design offers only a snapshot of stakeholder perspectives during the early phase of adoption and may not capture how attitudes, practices, or implementation challenges evolve over time. In addition, participants were asked to reflect retrospectively on their initial experiences with the technology, which may have introduced recall bias. Several challenges discussed by participants, including interpretation of CAD outputs and threshold-based referral decisions, were primarily described as occurring during the early implementation period and may not reflect routine practice at the time of data collection.

Finally, although the study sought to incorporate diverse perspectives from multiple stakeholder groups and data sources, it was limited to a single geographic setting and project context. While these factors support the credibility of the findings, the transferability of the results to other settings should be given caution, given differences in health systems, implementation models, and population contexts. Future research across multiple implementation sites and populations would help assess the extent to which these findings are transferable to other TB screening programmes.

## Conclusion

This study demonstrates that implementing CAD in TB active case finding is both feasible and acceptable when supported by careful planning and adaptation to local contexts. CAD integration was perceived to improve screening efficiency and capacity, optimize workflows, and provide valuable decision support. However, successful implementation reportedly requires robust technical infrastructure, clear data management protocols, and investments in training to build user confidence. Community participation was influenced by perceived healthcare needs, trust in technology, and process simplicity, underscoring the importance of communication and engagement. Overall, CAD offers significant potential to strengthen TB screening, particularly in resource-limited settings, provided that implementation strategies address technical, operational, and social dimensions. Sustainable implementation hinges not only on technical support but also on the meaningful involvement of healthcare workers and communities, whose insights are crucial for ensuring ethical and effective digital health innovation. The experience in Tondo offers valuable lessons, highlighting both the challenges and opportunities of deploying CAD tools in high-burden, resource-constrained settings. These insights can guide future implementations, helping tailor approaches that are both locally responsive and globally informed

## Supporting information

S1 Checklist Inclusivity in global research. (DOCX)

S2 Checklist Consolidated criteria for reporting qualitative studies (COREQ): 32-item checklist. (DOCX)

S1 Tools Qualitative topic guide and data collection instruments. (DOCX)
