# Supplementary material for: Implementing computer‑aided detection for TB active case finding: A qualitative study of feasibility and acceptability in Tondo, Manila
Source: PLOS Glob Public Health. 2026 Oct 8;6(10):e0007429. doi: 10.1371/journal.pgph.0007429 (PMC13649030; doi:10.1371/journal.pgph.0007429)
Supplement: Supplementary file 2 [file pgph.0007429.s002.docx]

**Consolidated criteria for reporting qualitative studies (COREQ): 32-item checklist**

**MANUSCRIPT TITLE: Feasibility and acceptability of the implementation of computer-aided detection software in TB active case finding: A qualitative study in Tondo, Manila.**

| **No. Item** | **Guide questions/description** | **Response** |
| --- | --- | --- |
| **Domain 1: Research team and reflexivity** |  |  |
| *Personal Characteristics* |  |  |
| 1. Interviewer/facilitator | Which author/s conducted the interview or focus group? | In the context of Tondo, decisions about interviewer assignment were made to minimize power imbalances and ensure participant comfort. Emelie Yonally Phillips, an American of Western European descent, conducted interviews with MSF healthcare staff, Physicians-in-Charge of HCs, and National TB Program staff. John Carlo Palmado and Carmina Duyala, research assistants of Filipino descent with contemporary ties to Manila, led activities with community leaders and ACF participants, with gender matching applied where appropriate to foster trust and comfort. |
| 2. Credentials | What were the researcher’s credentials? (E.g.  PhD, MD) | Valentina Carnimeo, MSc, MPH  Emelie Yonally Phillips, MPH |
| 3. Occupation | What was their occupation at the time of the study? | Valentina Carnimeo was a senior epidemiologist for Epicentre/MSF. Emelie Yonally Phillips was a senior research consultant for Epicentre/MSF. John Carlo Palmado and Carmina Duyala were hired for this project as MSF Philippines research assistants. Sheryl Peral was hired for this project as a translator for MSF Philippines. |
| 4. Gender | Was the researcher male or female? | Valentina Carnimeo, Emelie Yonally Phillips, Carmina Duyala, and Sheryl Peral (females)  John Carlo Palmado (male) |
| 5. Experience and training | What experience or training did the researcher have? | Valentina Carnimeo is a senior epidemiologist with Epicentre/MSF with 8 years leading research projects globally. Emelie Yonally Phillips is a senior mixed methods researcher with 8 years leading qualitative research studies for humanitarian projects. John Carlo Palmado is a development worker and social researcher with experience working in local and international non-government organizations. Sheryl Peral works for the Philippine government under the Department of the Social Welfare and Development, has wide experience in qualitative and quantitative research projects, and is experienced in professional translation. |
| *Relationship with*  *participants* |  |  |
| 6. Relationship established | *Was a relationship established prior to study commencement?*  . | No prior relationship was established between the researchers and participants before the commencement of the study. |
| 7. Participant knowledge of the interviewer | *What did the participants know about the researcher? (e.g. personal goals, reasons for doing the research).* | During the informed consent process each researcher introduced themselves, their role in the study, and disclosed that while they were affiliated with MSF, they were not directly linked to the Tondo TB project and the goal of their work as a researcher was to understand the experiences and perspectives of the participants. |
| 8. Interviewer characteristics | *What characteristics were reported about the interviewer/facilitator? (e.g. Bias, assumptions, reasons and interests in the research topic)* | While all researchers were affiliated with either Epicentre or MSF, they were hired specifically for this research project and had no prior or post affiliation with the MSF ACF project. All aimed to maintain a neutral stance, centering the participants’ voice, with a motivation to improve programming and TB outcomes in Tondo. We acknowledge that our identities and affiliations may have influenced interactions and interpretation. To mitigate bias, we engaged in reflexive practices, including team discussions on assumptions, end of data collection day debriefs, and triangulation of data sources, to ensure findings reflect participants’ perspectives. |
| **Domain 2: Study design** |  |  |
| *Theoretical framework* |  |  |
| 9. Methodological orientation and Theory | *What methodological orientation was stated to underpin the study? (e.g. grounded theory, discourse analysis, ethnography, phenomenology, content analysis).* | The study was underpinned by a Framework Analysis approach. The Unified Theory of Acceptance and Use of Technology (UTAUT) provided the analytical framework to guide both data collection and analysis. Themes were deductively mapped to UTAUT domains (performance expectancy, effort expectancy, social influence, and facilitating conditions), while allowing inductive subthemes to emerge from the data. |

| *Participant selection* |  |  |
| --- | --- | --- |
| 10. Sampling | *How were participants selected? (e.g. purposive, convenience, consecutive, snowball)* | Non-probability sampling methods were used to identify participants. Community leaders were identified by the Barangay Chairperson and recruited to participate in the FGDs. Purposive quota sampling was used to identify ACF participants in person at the end of screening by the exit registrar nurse using quota of gender and CAD score above and below threshold, with efforts made to include ACF participants of various ages and those recruited to the ACF site through community mobilization and household contact tracing. The Physicians-in-Charge of the four HCs in which MSF ACF activities had been conducted at the time of the study were recruited into the study in person. Purposive sampling was used to identify members of the MSF Tondo project managers and staff and the MHD TB Control Program that would be able to provide the most information about the acceptability and feasibility of implementing CAD into this project and context. |
| 11. Method of approach | *How were participants approached? (e.g. faceto-face, telephone, mail, email)* | All participants with the exception of one MSF staff member were approached face-to-face. The MSF staff member who was off-site at the time of the study was approached via email. |
| 12. Sample size | *How many participants were in the study?* | The study included a total of 50 participants. 8 MSF project managers and staff, 4 Physicians-in-Charge, 3 MHD TB control programme staff, 19 community leaders, and 16 ACF participants. |
| 13. Non-participation | *How many people refused to participate or dropped out? Reasons?* | All staff, Physicians-in-Charge, and MHD TB Control Program staff invited agreed to participate. No record was kept of community leaders and ACF participant refusals. However, the reported reason cited by those who refused was time constraints. |
| *Setting* |  |  |
| 14. Setting of data collection | *Where was the data collected? (e.g. home, clinic, workplace)* | Data was collected at the local barangay community center, ACF sites, MSF project office, Manila TB Control Office, and via Zoom. |
| 15. Presence of nonparticipants | *Was anyone else present besides the participants and researchers?* | No. Although the focus group discussions with community leaders in the local barangay community center did encounter interruptions by community members a few times. At these times the discussion would be halted until the community members left, the doors were closed, and the room contained only participants and researchers once again. |
| 16. Description of sample | *What are the important characteristics of the sample? (e.g. demographic data, date)* | Demographic data such as age, gender, educational level, household size, and barangay were recorded at the beginning of each FGD with community leaders and IDI with ACF participants. CAD score above or below the threshold and referral through mobilization or contact tracing were also recorded for ACF participants. Position and time in the position were recorded for MSF staff, Physicians-in-Charge, and MHD TB control programme staff to understand experience. |
| *Data collection* |  |  |
| 17. Interview guide | *Were questions, prompts, guides provided by the authors? Was it pilot tested?* | Semi-structured FGD and IDI guides tailored to each study participant group were developed to explore each aspect of the UTAUT framework. UTAUT posits that the acceptance and use of technology is informed by performance expectancy, effort expectancy, social influence and facilitating conditions.  All tools were pilot tested prior to data collection. The qualitative research team conducted pilots in a barangay that was not included in the study in which ACF activities had been conducted prior to the implementation of CAD. A pilot FGD was held with community leaders and two pilot ACF participant IDIs conducted with community members. The IDIs for MSF staff, Physicians-in-Charge, and MHD staff were piloted with MSF project staff that were not recruited into the study. |
| 18. Repeat interviews | *Were repeat interviews carried out? If yes, how many?* | No repeat interviews were conducted. |
| 19. Audio/visual recording | *Did the research use audio or visual recording to collect the data?* | Audio recording was used to document all IDIs and FGDs with participants' consent to ensure accuracy in analysis. No visual recording was conducted during the study. |
| 20. Field notes | *Were field notes made during and/or after the interview or focus group?* | During and directly following each FGDs and IDIs, field notes were written by the researcher who conducted the activity to capture emerging themes, record observations of the setting and interaction, and monitor data saturation. |
| 21. Duration | *What was the duration of the inter views or focus group?* | Each focus group discussion lasted approximately 90 minutes. Individual interview lasted approximately 30-60 minutes, depending on the level of participant engagement and the depth of discussion. |
| 22. Data saturation | Was data saturation discussed? | Data saturation was considered a key criterion for determining the completion of sampling and discussed during daily debriefings. |
| 23. Transcripts returned | *Were transcripts returned to participants for comment and/or correction?* | Transcripts were not returned to participants for review. |
| **Domain 3: analysis and findings** |  |  |
| *Data analysis* |  |  |
| 24. Number of data coders | How many data coders coded the data? | While preliminary analysis was done by the entire qualitative team, data coding was conducted by a single researcher. This maintained consistency in coding, while ensuring the broader analysis benefited from multiple perspectives. |
| 25. Description of the coding tree | *Did authors provide a description of the coding tree?* | The authors did not explicitly provide a description of the coding tree. However, the themes, sub-themes, and associated UTAUT components were provided in a matrix to elucidate the analysis. |
| 26. Derivation of themes | *Were themes identified in advance or derived from the data?* | A combination of inductive and deductive approaches was used in the analysis. Initially all qualitative data were analysed with inductive open coding to explore and consider all possible meanings, and an initial coding scheme was developed. Following this open, descriptive coding, codes were organized into sub and parent-codes. Parent codes were further grouped into themes connected to the relevant components of the UTAUT framework and checked against the pre-liminary findings and themes noted by JCP and CD. |
| 27. Software | *What software, if applicable, was used to manage the data?* | NVivo 13 software |
| 28. Participant checking | *Did participants provide feedback on the findings?* | Although participants did not formally validate the findings, analytic rigor was maintained by anchoring themes in participants’ own words and conducting a thorough, systematic thematic analysis to ensure faithful representation of their perspectives. |
| *Reporting* |  |  |
| 29. Quotations presented | *Were participant quotations presented to illustrate the themes/findings? Was each quotation identified? (e.g. participant number)* | Participant quotations were provided to illustrate themes and identified by participant type. As many of the target populations are small (MSF project staff, Physicians-in-Charge, MHD TB control program staff) or participation was well known (community leaders, ACF participants), it was determined that participant numbers should not be included. |
| 30. Data and findings consistent | *Was there consistency between the data presented and the findings?* | Yes. There was clear alignment between the data and the findings. Each thematic interpretation was directly supported by participant narratives, and illustrative quotations were used to demonstrate how the analysis was derived from the data. |
| 31. Clarity of major themes | *Were major themes clearly presented in the findings?* | Major themes were clearly presented in the findings, with sub-themes explored and UTAUT components identified. |
| 32. Clarity of minor themes | Is there a description of diverse cases or discussion of minor themes? | Yes. The analysis included descriptions of diverse participant experiences, and minor themes were noted where relevant to provide additional contextual understanding. |
