# Supplementary material for: Implementing computer‑aided detection for TB active case finding: A qualitative study of feasibility and acceptability in Tondo, Manila
Source: PLOS Glob Public Health. 2026 Oct 8;6(10):e0007429. doi: 10.1371/journal.pgph.0007429 (PMC13649030; doi:10.1371/journal.pgph.0007429)
Supplement: Supplementary file 3 [file pgph.0007429.s003.docx]

S1 Tools. Qualitative Topic Guide and Data Collection Instruments

## A. Topic Guide

A topic guide is a thematic framework designed to help prioritise what themes will be excluded or included in the research tools. A topic guide acts as an overview of the key sections for research. Research tools for relevant stakeholder groups will be developed from the topic guide and will be tailored to each specific group being interviewed.

The topic guide was built out using the Unified Theory of Acceptance and Use of Technology (UTAUT) as the conceptual framework for understanding user and patient acceptance and adoption of ACF with CXR/CAD in Tondo. UTAUT posits that the acceptance and use of technology is informed by performance expectancy, effort expectancy, social influence and facilitating conditions. Each of these determinants of acceptance and use can be moderated by factors such as gender, age, experience, and voluntariness of use. Use of this framework allows for the exploration of various factors at individual, community, organizational, and policy levels that influence user, physician, and patient acceptance and use of digital X-ray and CAD4TB.

**Fig 1.** The Unified Theory of Acceptance and Use of Technology

**Knowledge:** The knowledge and sources of information participants have about digital Xray and CAD usage and procedures for active case finding and TPT initiation

- Knowledge about digital Xray and CAD usage for active TB case finding
- Knowledge about screening/referral process
- Perceived comprehensiveness of training/education materials
- Context and sources of information

**Performance Expectancy:** The capability of digital Xray and CAD use to provide benefits and enhance the performance of active TB case finding and TPT initiation according to the participants expectations

- Perceived usefulness of digital Xray and CAD for TB screening/for TB diagnosis/for treatment follow up
- Perceived process fit/how does the process for digital Xray and CAD fit with user/patient expectations for process flow/fit within daily activities/scope of work
- Perception of service providers (e.g. competency, ability, trustworthinesss)
- Perceived relative advantage of digital Xray and CAD over standard practices/types of TB screening
- Outcome expectations/trust in outcomes/challenges/solutions

**Effort Expectancy:** Expectations about the ease of use of digital Xray and CAD for active TB case finding and TPT initiation

- Perceived ease of use of the digital Xray and CAD equipment/of the outputs/of the screening process/of the referral process/perceived workload/time commitment
- Complexity of using digital Xray and CAD/of understanding the outputs/of the screening process/of the referral process
- Self-efficacy/steps of the testing procedure at risk of errors/recommendations for further training, supervision, explanation/perceived barriers

**Social Influence:** The expected influence of others on the user to start and continue using the technology

- Social and organisational norms surrounding TB screening/diagnosis/treatment
- Social and organisational norms surrounding AI usage
- Gender roles

**Behavioral intention:** The expectation of the user/patient’s intention to perform plans and decisions regarding the use of digital Xray and CAD

- Intentions and attitudes around the continued use/expanded use of digital Xray and CAD for active TB case finding/trust in the technology/results/health system
- Intentions and attitudes regarding referral/treatment/TPT initiation

**Facilitating conditions:** The expected level of organizational and technical infrastructure that can support the use of digital Xray and CAD for active TB case finding and TPT initiation

- Perceptions about the level of organisational infrastructure that exists to support the use of digital Xray and CAD for active TB case finding in the study site/ease and challenges of set up
- Perceptions about the level of technical infrastructure that exists to support the use of digital Xray and CAD for active TB case finding in the study site/ease and challenges of implementation
- Perceptions about the policy level enabling environment for the use of digital Xray and CAD for active TB case finding in the study site
- Coordination and integration with other services
- Barriers to provision/uptake of services (e.g. access/availability/staffing/time/logistics)

## B. In-Depth Interview Guide: MSF Staff

ID CODE: _______________

FACILITATOR/NOTE TAKER: _________________

LOCATION: ___________________

TIME: ____________________

**Knowledge:** The interview will begin with some questions exploring the user knowledge and about digital Xray and CAD4TBfor active TB case finding. The aim is to assess user understanding of the technology and their perceptions of the training and education they have been provided.

- Can you tell me what you know about digital Xray and computer aided diagnostic software (called CAD4TB)?
  - Have you heard of using CAD4TBfor TB screening before this project?
  - Have you used AI in your job prior to this project?
- Can you describe the training your received for using digital Xray and CAD4TB for TB screening?
  - Did you find the training/information provided to be comprehensive?
  - Are there areas and/or topics you would have liked further training/information on?
  - Are there any ways you think the training/information could be improved?
- Can you describe the screening and referral process for patients with TB?
  - What are the steps that are taken before patients get to you?
  - What are the steps you take a patient through?
  - What are the next steps thereafter?

**Performance/Effort Expectancy:** This section of the interview will explore the user's perceptions and expectations about ease of use of digital X-ray and CAD4TB, and its capability for providing benefits and enhance the performance of active TB case finding and TPT initiation. The aim is to understand the software efficiency and usability from the perspective of the users.

- Can you tell me about your experience using digital X-ray and CAD4TB to screen patients for TB?
  - What is the process like?
  - Is it easier or harder than you expected it would be? In what ways?
- Can you describe any challenges you face in performing the digital X-ray? In using the CAD4TB? In interpreting the results? How did you deal with those challenges?
  - Are there steps in the testing procedure that you think have high risk for errors? How do you work to minimize these errors?
  - Can you describe any benefits you have experienced from the use of CAD4TB? Has it empowered you in your diagnosis? Added to your knowledge base?
  - Can you tell me how comfortable you feel interpreting the results and providing recommendations/referrals?
  - How comfortable are you in explaining the process and results to your patients? Do you feel you are able to explain fully in a way they understand and accept?
- Can you describe how well the process fits into your workflow?
  - Are there aspects of the process that work well for you or aspects that do not work well?
  - Are there changes you would recommend for how to integrate digital Xray and CAD4TB into your daily activities?
- Can you describe how well active case finding using digital Xray and CAD4TB fit into the TB screening algorithm?
  - How does it compare to the previous system (human reading)?
  - Do you think digital X-ray and CAD4TB is being used in the right place in the algorithm to make the largest impact? If not, where in the diagnostic algorithm do you think digital Xray and CAD4TB are most useful?
- Can you describe how useful you find the use of digital Xray and CAD4TB for screening of TB?
  - How accurate/sensitive do you think the screening is compared to other tools?
  - Can you describe any change in the ability to screen for TB from using this software?
  - In your experience, do your patients accept and trust the CAD4TB results?
- How useful do you find digital Xray and CAD4TB for conducting active case finding in a population?
  - Is it useful for screening large quantities of people?
  - For mobile or door to door screening?
  - How does it compare to other tools for TB screening?
- Can you describe any impact you have perceived of using digital Xray and CAD4TB on referral follow up and/or treatment or potential TPT initiation?
  - Has a digital Xray and CAD4TB screening increased or decreased the likelihood that patients follow up for treatment initiation? If so, why do you think this happens?

**Social Influence:** The questions in this section explore social and cultural norms and attitudes at the community and organisational level regarding the use of AI in health care. The aim is to understand the expected influence of others on the acceptance and use of digital Xray and CAD$TB.

- Can you describe how the community views or reacts to the use of digital Xray and AI in healthcare?
  - Is there a culture of acceptance of new technology? Distrust or concerns?
  - Are there any social or cultural practices or beliefs that impact the acceptance of AI?
- Can you describe how other health care workers you interacted with respond to the introduction of AI into health care?
  - Is there an organisational cultural of acceptance of or resistance to new technologies?
  - Are there any concerns health workers using CAD4TB have about the introduction of AI?
  - Is this the same for different groups of health care workers? Nurses? Doctors? Lab staff?

**Behavioural intention:** The questions in this section explore user intentions and plans regarding the use of digital Xray and CAD4TB.

- Do you think that digital Xray and CAD4TB should continue to be used for TB screening in Tondo?
  - Are there any changes to the project or process that you have or intend to make?
- Do you have any ideas or plans going forward regarding the use of digital Xray and CAD4TB?
  - Intentions to keep/stop/change usage?
  - Advocating for expanded use? Advocating against use?
  - Changing the locations in the diagnostic algorithm?
  - Learning more?
  - Changes to the patient education?

**Facilitating conditions:** The questions in this section explore user perceptions around the expected level of organizational and technical infrastructure that can support the use of digital Xray and CAD4TB for active TB case finding.

- Can you describe any barriers you have experienced to the provision of TB screening with digital Xray and CAD4TB?
  - Staffing/time/logistics
- Can you describe and barriers you have seen or heard about to patient uptake of these services?
  - access/availability
- Can you tell me about the level of organisational infrastructure that exists to support the use of digital Xray and CAD4TB for active TB case finding in the study site?
  - Can you describe the organisational set up?
  - Have there been organisational challenges with implementation? How were these overcome?
  - What enabling factors helped with setting up the program in Tondo?
  - Do you think these same challenges and enabling factors exist in other areas that have high risk of TB?
  - Do you have recommendations for improvement?
- Can you tell me about the level of technical infrastructure that exists to support the use of digital Xray and CAD4TB for active TB case finding in Tondo?
  - Can you describe the logistics of setting up the technical infrastructure?
  - Have there been technical and logistical challenges with implementation? How were these overcome?
  - What have been the key facilitating technical and logistical factors that have helped with implementation?
  - Do you think these same challenges and facilitating factors exist in other areas at high risk of TB?
  - Do you have recommendations for improvement?
- Can you tell me about anything at the policy level that has helped or impeded implementation?
- Can you describe how this programme has been coordinated and integrated with other relevant services?
  - Do you think this has been done effectively?
  - Do you have recommendations for improvement?
- What do you think about the use of digital Xray and CAD4TB for TB screening in other areas?
  - Rural areas?
  - Other countries with high rates of TB?
  - Passive case finding?

**Closing questions:** In this section, patients are asked to discuss anything more they think is pertinent and open the discussion for their questions. The aim is to highlight any missed areas of relevance/importance and allow patients to ask questions as well.

- What do you feel have been the most important things that we have spoken about? (recap key points)
- Is there anything else that you would like to discuss?
- Is there anything specific that you think we should be asking patients/service providers in our discussions with them?
- Do you have any questions for us?

**Thank you and description of expected timeline and next steps for reporting.**

## C. In-Depth Interview Guide: ACF Participants

The interview guide tool is meant to provide a loose framework exploring the key thematic areas related to the research aims. As these are intended to be semi-structured interviews, participants are encouraged to lead the discussion. The interview guide tool will help ensure that all key topics will be discussed, however, the order of questions presented in this guide need not be strictly adhered to. Additionally, the probing questions included in this guide should be understood as suggestions for deeper exploration of topics. These probes have emerged from the literature, however, in the field it is possible that not all will turn out to be relevant. Conversely, participants may introduce new relevant information and areas that interviewers will then explore further through naturally emerging probing questions.

Demographic information to be collected on separate demographic information form

**Knowledge:** The interview will begin with some questions around patient’s knowledge and sources of information about TB, digital Xray and CAD4TB software, and the process of being screened and treated for TB. The aim is to gain a better understanding of what patients know, their sources of information, their satisfaction and trust in the information they have, and how this influences their acceptance and intended use of digital Xray and CAD4TB TB results.

- Can you tell me about TB in your community?
  - Is it common/rare?
  - What are the symptoms of TB?
  - How is TB transmitted to another person?
  - Who is most at risk of TB?
  - If a person in your community thinks he/she was infected with TB, what do they usually do?
  - Where do you get information about TB?
  - Do people in your community go for TB screening/treatment?
- Can you tell me what you know about digital chest Xrays and computer aided diagnostic software for TB (called CAD4TB)?
  - How did you learn about CAD4TB?
  - What information were you given?
  - How satisfied are you with the information provided?
  - What do you think is the best way for people in your community to get information about digital Xray and CAD4TB for TB screening? Who and what are the most trusted sources?
- What are the steps a person must go through to be screened for TB? Once they have their CAD4TB results, what are the next steps?

**Performance Expectancy/Effort Expectancy:** Next the interview explores the patients experience and expectations around the process of TB screening and the use of digital Xray and CAD4TB. The aim here is to understand patient expectations and satisfaction around the benefits and outcomes of digital Xray and CAD4TB ,the set up and process of screening and referrals.

- Can you tell me about your experience being screened for TB with the digital Xray and CAD4TB?
  - Have you ever been screened for TB before today? If yes, what was the process like?
  - What was the process like (referring to screening with CAD4TB)? Was it harder or easier than you expected it would be?
  - Can you describe any challenges you had in going for screening? During the process?
  - Were there things about the process that you liked or did not like?
  - Are there things that you would have liked done differently?
- Can you tell me about your experience with the service provider that screened you for TB?
  - Were there things you liked or did not like about your experience with the service provider?
  - How well do you think they explained what computer assisted diagnostic software (CAD4TB) is and what the results from the X-ray and software meant?
  - How well do you think they explained what you should do next after getting the results?
  - Do you think people in your community trust these service providers? Why or why not?

How do you feel about having a machine determine whether or not you should be further tested for TB instead of a radiologist/doctor?”

- Can you tell me about what you were told about CAD4TB during the screening process?
  - How was the CAD4TB software explained to you? Were you told that it was artificial intelligence software that examines the X-ray?
  - How were your results explained to you? Are the results easy to understand? Why or why not?
- Can you tell me about your thoughts on how useful digital Xray and CAD4TB are for telling if a person is or isn’t at risk for TB?
  - Do you trust the CAD4TB results? Why or why not?
  - Do you have concerns for using digital Xrays and CAD4TB software to screen for TB?
  - If you have been screened for TB before (for example with a sputum test), which process for screening did you prefer? Which results did you trust more?
- Can you tell me about your thoughts on the screening and referral process?
  - Is it accessible for people in your community?
  - If you have been screened before, is this screening process better or worse than how TB screening used to be done?
  - Is there anything that could improve the screening and referral process?

**Social Influence:** This section of the interview explores questions around community norms and perceptions of TB and decision making. The aim is to gain understanding of what sociocultural factors influence the acceptance and use of TB screening activities.

- Within your family, who makes decisions about going to the health facilities?
  - About TB screening?
  - About treatment for TB?
  - Is this typical for families in your community?
- How do people in your community feel about TB?
  - Is TB a big deal in Tondo?
  - Are people in the community open about having TB? Why or why not?
  - Is TB screening a priority for people here?
  - Is TB treatment a priority for people here?
  - Do you think people in the community think that active case finding with digital Xray and CAD4TB will be beneficial to the people?
- Can you tell me how people in your community respond to new AI technologies like CAD4TB being used for health care?
  - Do people in your community usually accept new technologies easily?
  - Are there any concerns people have about the use of AI for diagnosis?
  - Are there any social or cultural practices or beliefs that impact the acceptance of AI?

**Behavioural intention:** The following questions explore the patient’s intention to act on digital Xray and CAD4TB results and intention to use and recommend digital Xray and CAD4TB for future TB screenings.

- If you experience TB symptoms in the future, what would you do?
  - Would you go for screening again at health facility or to a general screening? CAD4TB or other?
- If someone in your family experiences TB symptoms, what would you do?
  - Would you take them or recommend they go for TB screening with CAD4TB?
- Would you recommend that other people in your community go for the digital Xray and CAD4TB TB screening? What influences your decision to recommend or not?

**Closing questions:** In this section, patients are asked to discuss anything more they think is pertinent and open the discussion for their questions. The aim is to highlight any missed areas of relevance/importance and allow patients to ask questions as well.

- What do you feel have been the most important things that we have spoken about? (recap key points)
- Is there anything else that you would like to discuss?
- Is there anything specific that you think we should be asking patients/service providers in our discussions with them?
- What do you think is the best way to share the results of this research back to your community? We may not be able to share the results in the way that you suggest, but we will use everyone’s recommendations to try to find a way that is good for the community to share the results back.
- Do you have any questions for us?

**Thank you and description of expected timeline and next steps for community reporting.**

## D. In-Depth Interview Guide: Physicians in Charge

ID Code: ____________________

FACILITATOR/NOTE TAKER: ________________________

LOCATION: _______________________

TIME: ___________________________

DATE: __________________________

**Knowledge:** The interview will begin with some questions exploring physicians’ knowledge about digital Xray and CAD4TB for TB screening. The aim is to assess physician understanding of the technology and their perceptions of the information they have been provided.

- Can you tell me what you know about MSF?
  - The work they are doing here in Manila?
- Can you tell me what you know about digital Xray and CAD4TB software?
  - Have you heard of using CAD4TB for TB screening before this project?
  - Have you used AI in your job prior to this project?
- Can you describe the information you have been provided with for using digital Xray and CAD4TB for TB screening?
  - Did you find the information provided to be comprehensive?
  - Are there areas you would have liked additional information?
  - Are there any ways you think the information provided could be improved?
- Can you describe the screening and referral process for TB?
  - Were the steps clear to you before starting?
  - What are the steps that are taken before patients get to you?
  - What are the steps you take a patient through?
  - What are the next steps?

**Performance/Effort Expectancy:** This section of the interview will explore physician perceptions and expectations about the capability of digital Xray and CAD4TB use to provide benefits and enhance the performance of TB case finding and TPT initiation and the ease of use of the CAD4TB outputs. The aim is to understand the perspectives of the physicians’ who are making diagnostic and treatment decisions based on CAD4TB outputs and referrals.

- Can you tell me about your experience with MSF on this project?
  - Is the project coordinated with the Health Center?
  - Is it integrated into the work of the Health Centre?
  - How do you feel about the level of collaboration? Compared to other organizations?
  - Are they ways to improve integration/coordination?
- Can you tell me about your experience having patients referred after being screened for TB with digital Xray and CAD4TB?
  - What is the process like?
  - How do the CAD4TB outputs impact the way you diagnose and treat patients?
  - Can you tell me how comfortable you feel making decisions about further testing, diagnosis, and treatment?
  - Can you describe any benefits you have experienced from the use of CAD4TB? Added to your knowledge base?
  - Do you feel you are able to explain fully in a way they understand and accept?
- Can you describe how well the use of CAD4TB screening fits into your workflow?
  - Are there aspects of the referral process that work well for you or aspects that do not work well?
- Can you describe how well active case finding using digital Xray and CAD4TB fit into the TB screening algorithm?
  - How does it compare to the previous system?
  - Do you think digital Xray and CAD4TB is being used in the right place in the algorithm to make the largest impact? If not, where in the screening algorithm do you think digital Xray and CAD4TB are most useful?
- How useful do you find digital Xray and CAD4TB for conducting active case finding in a population?
  - What do you think the impact will be of using digital Xray and CAD4TB for TB screening in Tondo?
  - How does it compare to other tools for TB screening?
- Can you describe any impact you have perceived of using digital Xray and CAD4TB on referral follow up and/or treatment or TPT initiation?
  - Has a digital Xray and CAD4TB screening increased or decreased the likelihood that patients follow up? If so, why do you think this happens?

**Social Influence:** The questions in this section explore social and cultural norms and attitudes at the community and organisational level regarding the use of AI in health care. The aim is to understand the expected influence of others on the acceptance and use of digital Xray and CAD4TB.

- Can you describe how patients in the community view or react to the use of AI in screening?
  - Is there a culture of acceptance of new technology? Distrust or concerns?
  - Are there any social or cultural practices or beliefs that impact the acceptance of AI?
- Can you describe how other health care workers you interact with respond to the introduction of AI into screening?
  - Is there an organisational cultural of acceptance of or resistance to new technologies?
  - Are there any concerns health workers have about the introduction of AI?
  - Is this the same for different types of health care workers?

**Behavioral intention:** The questions in this section explore user intentions and plans regarding the use of digital Xray and CAD4TB.

- Do you think that digital Xray and CAD4TB should continue to be used for TB screening in Tondo?
  - Are there any changes to the process that you have or intend to make?
- Do you have any ideas or plans going forward regarding the use of digital Xray and CAD4TB?
  - Intentions to keep/stop/change usage?
  - Advocating for expanded use? Advocating against use?
  - Learning more?
  - Alterations to the patient education?

**Facilitating conditions:** The questions in this section explore user perceptions around the expected level of organizational and technical infrastructure that can support the use of digital Xray and CAD4TB for active TB case finding.

- Can you describe any barriers you have experienced to the provision of referred follow-up for patients screened for TB with digital Xray and CAD4TB?
  - Are there barriers to getting TB confirmation tests or treatment for those who have a positive CAD4TB output?
  - Are there barriers to TPT provision for those referred?
- Can you describe any barriers you have seen or heard about to patient uptake of these services?
  - Any barriers on the patient side for going for follow up referral? Accessibility/availability/stigma?
  - Any barriers for the patient for initiating treatment or TPT? Cost/travel/stigma/availability?
- Can you tell me about the level of organisational infrastructure that exists to support patient referrals from active TB case finding in the study site?
  - Can you describe the organisational set up?
  - Have there been organisational challenges with accommodating referrals? With incorporating digital Xray and CAD4TB results into diagnostic and treatment decisions? How were these overcome?
  - What enabling factors helped?
  - Do you have recommendations for improvement?
- Can you tell me about the level of technical infrastructure that exists to support the use of digital Xray and CAD4TB for active TB case finding in Tondo?
  - Can you describe the logistics of setting up the technical infrastructure for using Xray and CAD4TB results in diagnostic and treatment decisions?
  - Have there been technical and logistical challenges with implementation? How were these overcome?
  - What have been the key facilitating technical and logistical factors that have helped with implementation?
  - Do you have recommendations for improvement?
- Can you tell me about anything at the policy level that has helped or impeded implementation?
- Can you describe how this programme has been coordinated and integrated with other relevant services?
  - Do you think this has been done effectively?
  - Do you have recommendations for improvement?
- What do you think about the use of digital Xray and CAD4TB for TB screening in other areas?
  - Rural areas?
  - Other countries with high rates of TB?
  - Passive case finding?

**Closing questions:** In this section, participants are asked to discuss anything more they think is pertinent and open the discussion for their questions. The aim is to highlight any missed areas of relevance/importance and allow patients to ask questions as well.

- What do you feel have been the most important things that we have spoken about? (recap key points)
- Is there anything else that you would like to discuss?
- Is there anything specific that you think we should be asking patients/service providers in our discussions with them?
- Do you have any questions for us?

**Thank you and description of expected timeline and next steps for reporting.**

## E. In-Depth Interview Guide: Key Stakeholders

ID CODE:

ACTIVITY:

PARTICIPANTS:

FACILITATOR:

DATE:

LOCATION:

**Background:** The interview will begin with some questions exploring the key stakeholders position and role in the planning/preparation and implementation of the MSF TB ACF programme with chest X-ray and CAD4TB4TB. The aim is to understand the position, expertise, involvement and responsibilities of the stakeholder in reference to the programme.

- Can you tell me about your role with your organization?
  - How long have you been at your current role?
  - What are your main job duties?
- Can you tell me about your involvement with the MSF TB ACF program in Tondo?
  - When did your involvement with the program begin?
  - What role/objectives did/do you have in the program set up and implementation?

**Programme planning/preparation:** This section of the interview will ask questions about the planning/preparation of the MSF TB ACF programme with chest X-ray and CAD4TB4TB. Planning/preparation includes initial proposal development, convening of stakeholders, approval processes, identification, sourcing, and calibration of equipment, etc. The aim is to document the process of planning/preparation, along with challenges, enablers and lessons learned.

- Can you tell me a bit about the planning/preparation of the program?
  - What were the initial steps that were taken?
  - Who was involved?
- What major decisions had to be made for the planning/preparation and implementation of ACF with chest X-ray and CAD4TB4TB software?
  - What decisions were made regarding the planning/preparation/design of ACF?
    - Who was involved? Why were the decisions made the way they were? What factors were considered when making these decisions?
  - What decisions were made regarding the planning/preparation of the mobile chest X-ray and the CAD4TB software?
    - Who was involved? Why were the decisions made the way they were? What factors were considered when making these decisions?
- What were the major challenges or obstacles to planning/preparation?
  - How were these obstacles overcome?
  - Can you describe if you think there are any ways these challenges may have been avoided?
- Can you tell me about the level of organizational infrastructure that exists to support the planning/preparation of ACF with chest X-ray and CAD4TB in Tondo?
- Can you tell me about the level of technical infrastructure that exists to support the planning/preparation for active TB case finding using chest X-ray and CAD4TB in Tondo?
  - Can you describe the planning/preparation of the logistics to set-up the technical infrastructure for using Xray and CAD4TB?
  - Have there been technical and logistical challenges with planning/preparation? How were these overcome?
  - What have been the key facilitating technical and logistical factors that have helped with planning/preparation?
  - Do you have recommendations for improvement?
- Can you tell me about anything at the policy level that has helped or impeded planning/preparation?

**Programme implementation:** This section of the interview will ask questions about the implementation of the MSF TB ACF programme with chest X-ray and CAD4TB. The aim is to document the process of implementation, along with challenges, enablers and lessons learned.

- Can you tell me a bit about the implementation of the program?
  - What was the process of rolling out the program?
  - Who was involved?
- What major decisions had to be made for the implementation of ACF with chest X-ray and CAD4TB software?
  - Were there any decisions made during the roll out of the programme that changed from what was planned during planning/preparation?
  - Who was involved? Why were the decisions made the way they were? What factors were considered when making these decisions?
- Can you tell me about the level of organizational infrastructure that exists to support the roll out of ACF with chest X-ray and CAD4TB in Tondo?
- Can you tell me about the level of technical infrastructure that exists to support the use of digital Xray and CAD4TB for active TB case finding in Tondo?
  - Can you describe the logistics of setting up the technical infrastructure for using Xray and CAD4TB results in diagnostic and treatment decisions?
  - Have there been technical and logistical challenges with implementation? How were these overcome?
  - What have been the key facilitating technical and logistical factors that have helped with implementation?
  - Do you have recommendations for improvement?
- What were the major challenges or obstacles to implementation?
  - How were these obstacles overcome?
  - Can you describe if you think there are any ways these challenges may have been avoided?
  - Are there barriers or challenges to implementation that persist?
- Can you tell me about anything at the policy level that has helped or impeded implementation?

**Scalability/replication:** This section of the interview will ask questions about the participants perspectives and recommendations regarding the scalability or replicability of the MSF TB ACF programme with chest X-ray and CAD4TB4TB to other contexts.

- Do you think that digital Xray and CAD4TB should continue to be used for TB screening in Tondo?
  - Are there any changes to the process that you have or intend to make?
- What would you describe as the necessary conditions/enablers for setting up ACF with chest X-ray and CAD4TB4TB software in other locations?
- What do you think about the use of digital Xray and CAD4TB for TB screening in other areas?
  - Rural areas?
  - Other countries with high rates of TB?
  - Passive case finding?

**Closing questions:** In this section, participants are asked to discuss anything more they think is pertinent and open the discussion for their questions. The aim is to highlight any missed areas of relevance/importance and allow patients to ask questions as well.

- What do you feel have been the most important things that we have spoken about? (recap key points)
- Is there anything else that you would like to discuss?
- Is there anything specific that you think we should be asking patients/service providers in our discussions with them?

## F. Focus Group Discussion Guide: Community Leaders

The focus group discussion guide is meant to provide a loose framework for facilitating discussions that explore the key thematic areas related to the research aims. As these are intended to group discussions, participants are encouraged to lead the discussion. The focus group discussion guide tool will help ensure that all key topics will be discussed, however, the order of questions presented in this guide need not be strictly adhered to. Additionally, the probing questions included in this guide should be understood as suggestions for deeper exploration of topics. These probes have emerged from the literature, however, in the field it is possible that not all will turn out to be relevant. Conversely, participants may introduce new relevant information and areas that facilitators will then explore further through naturally emerging probing questions.

Demographic information to be collected on separate demographic information form

Demographic information to be collected on separate demographic information form

PARTICIPANTS (GENERAL): _______________

MODERATOR: _______________

NOTE TAKER:

GROUP: _______________

DATE: _______________

TIME: _______________

PLACE: _______________

Introduction: Here the researcher(s) will introduce themselves and the study, explain the process and expectations of the focus group discussion, and retouch on consent and confidentiality

- Moderator(s) will introduce who they are and review the purpose of the study
- Review the process of the focus group, how it will be structured, and how long it will take.
- Review the plans for use for the data and informed consent and confidentiality
- Note that the focus group will be audio-recorded, and that participants’ identities will be kept confidential

Ground Rules: Ground rules and group norms are always established at the beginning of a focus group session to ensure mutual respect, consideration, and a supportive atmosphere for the discussion.

- All group members have a right to their viewpoints and opinions
- All group members have a right to speak without being interrupted or disrespected by other group members
- Group members will avoid dominating the conversation and will allow time for others to speak
- The moderator has the right to guide the timing and flow of the session topics but will allow the group to determine the importance and focus of the conversation, as appropriate
- Identities of group members will remain confidential; first names only will be used in labels and reference to one another during the session.
- Ask the group if they have any other ground rules that they would like to establish?

Ice Breaker (5-10 min): This ice breaker question is a chance to have each participant introduce themselves. Go around the circle and have each participant answer.

- Please share your first name and your leadership role in the community

Introductory Question (10 min): This question is intended to broadly introduce the topic of discussion and engage each participant in answering. Go around the circle and have each participant answer. This helps make participants more comfortable speaking and sets the tone for everyone contributing to the discussion.

- Please describe the health of your community in one sentence or a phrase

Transition Question: This question transitions more specifically into the topic that we are interested in while again engaging each participant in speaking. Go around the circle again to have each participant answer.

- How would you describe health care access and services in your community?

Discussion Questions: The following questions are intended to stimulate discussion among the participants regarding TB in the community, community health seeking behaviors, accessibility and acceptability of TB screening and health services in general and the ACF program in particular, and community acceptance and attitudes towards AI technologies in healthcare. Moderators should seek to facilitate and keep the discussion on track, but participants should be encouraged to discuss each topic among themselves. The listed probes are available to stimulate discussion as needed, and new probes may emerge from the discussion.

- How would you describe TB in your community?
  - Is TB common/rare?
  - What are the symptoms of TB?
  - How is TB transmitted to another person?
  - Who is at most risk of TB?
  - What do people in your community do if they have TB symptoms?
  - Who makes decisions about care seeking in families in your community?
  - Who is in charge of care provision in the home? Taking sick family members to the hospital? Getting medications?
  - Do you have specific examples or stories?
- Describe how people in your community feel about TB.
  - Is TB considered a serious issue in Tondo?
  - Are people in the community open about having TB? Why or why not?
  - Is TB screening a priority for people here?
  - Is TB treatment a priority for people here?
  - Do you have a specific story or example to share?
- How would you describe TB screening and TB health services in your community?
  - What services are available in your community? Are these sufficient?
  - What are the challenges people in your community face for getting diagnosed with TB? For accessing treatment? For accessing prevention medication (TPT)?
  - Describe the relationship between the community and the health workers? Is there trust established?
  - Do you have specific examples or stories of people in your community with TB screening and TB health services? Positive and/or negative examples?
- Describe the ways that the TB Active Case Finding impacts the community.
  - Do you have examples of community opinions or attitudes about the program?
  - Do you have examples or stories of people in your community who have been screened during the TB Active Case Finding? Can you tell us about their experience?
- Can you tell me how people in your community respond to new AI technologies like CAD being used for health care?
  - Do people in your community usually accept new technologies easily?
  - Are there any concerns people have about the use of AI for diagnosis?
  - Are there any social or cultural practices or beliefs that impact the acceptance of AI?
  - Can you give more details about that?
- Describe the potential impacts this program and technology could have on TB in your community.
  - Can you describe why you think these impacts will happen?
  - Would you recommend that other people in your community go for the digital Xray and CAD TB screening? Why or why not?
- Can you describe ways that TB screening and services can be improved to better support and care for people in your community?

**Closing questions:** In this section, participants are asked to discuss anything more they think is pertinent and open the discussion for their questions. The aim is to highlight any missed areas of relevance/importance and allow patients to ask questions as well.

- What do you feel have been the most important things that we have spoken about? (recap key points)
- Is there anything else that you would like to discuss?
- Is there anything specific that you think we should be asking patients/service providers in our discussions with them?
- What do you think is the best way to share the results of this research back to your community? We may not be able to share the results in the way that you suggest, but we will use everyone’s recommendations to try to find a way that is good for the community to share the results back.
- Do you have any questions for us?

**Thank you and description of expected timeline and next steps for community reporting**

## G. Observation Framework: ACF TB Screening Site

**Researcher:**

**Site:**

**Date:**

**Observation timeframe:**

**Facility organisation**

1. Describe where the digital X-ray and CAD4TB are performed
   a. How much space is needed to perform the tests?
   b. What equipment is present?
   c. How does the space feel? (cramped/organized/clean/private/open)
2. Describe where the CAD4TB is stored:
   a. Light:
   b. Temperature:
   c. Humidity:
3. Describe the patient flow through the site
   a. Are patients seen on a first come first serve or is there a list?
   b. How long does the process of screening a patient take from start of the screening to completion?
   c. Are there any observed bottlenecks in patient flow?
4. Describe the following aspects of the centre:

| Space available in centre |  |
| --- | --- |
| Privacy/confidentiality |  |
| Cleanliness of centre |  |
| Flow of service users |  |
| CAD4TB storage conditions |  |
| Data storage system |  |
| Adequate chairs/table |  |
| Posters/wall signs, leaflets |  |
| Electricity |  |
| Computer |  |
| Printer |  |
| Other, please specify……. |  |

**About the health care workers**

5. Who is performing the digital X-ray and CAD4TB?
   a. What is their background?
   b. Gender?
6. What other staff are present on the site?
   a. What are their roles?
   b. Gender of the staff:

**About the patients**

7. How many patients are on site during the observation time period?
8. Describe the patients present
   a. Age range:
   b. Genders (mostly men? Mostly women?):

**Patient/provider interaction:**

9. Describe the patient provider interaction:
   a. What information is given to the patient during the screening process?
   b. How much time is spent in explaining the screening process, the CAD4TB results, and the next steps for follow-up to the patient?
   c. Describe the tone/body language of the provider:
   d. Describe the communication style of the provider (respectful/kind tone/comprehensive/empathetic/short/cold):
   e. Describe the tone/body language of the patient (relaxed/comfortable/confused/stiff/distant):
   f. Describe the communication style of the patient (engaged/questioning/reserved/confused):
10. Describe any observed problems during the waiting or screening process:
    a. Were any concerns or confusion about the CAD4TB expressed?
    b. How did the provider respond to the problem?

**Other key observations/notes**
